# Feasibility, fidelity and initial effects of an app-based service for short-term antibiotic therapy: A pilot study in a primary care setting

**DOI:** 10.1016/j.rcsop.2026.100790

**Published:** 2026-04-21

**Authors:** Kirstin Messner, Vanessa Sutter, Samuel Allemann, Isabelle Arnet

**Affiliations:** a University of Basel, Department of Pharmaceutical Sciences, Basel, Switzerland; b TopPharm Hirschen Pharmacy, Magden, Switzerland

**Keywords:** Medication adherence, Community pharmacy, Antibiotics, Primary care, Mobile health

## Abstract

**Background:**

Short-term antibiotic treatments show medication non-adherence rates around 40%, limiting therapy success and increasing risk for recurring infections. Smartphone applications offer simple solutions to support patients' medication adherence.

**Objective:**

To evaluate service and trial feasibility of a smartphone-based adherence support delivered by community pharmacists and to explore initial effects.

**Methods:**

We conducted a cluster-randomized pilot trial. Community pharmacies recruited eligible patients with newly prescribed amoxicillin/clavulanic acid therapy when dispensing the prescription. Participants used an app to confirm medication intakes. The intervention group received intake reminders and text messages. We evaluated feasibility and fidelity based on data quality, participant satisfaction, and extent to which service activities were delivered as planned. For exploratory analyses, we used the Mann-Whitney *U* test, and calculated effect sizes.

**Results:**

Fifty-seven community pharmacies and 34 patients participated in the study (17 per group). Twenty-nine participants (85%) fully completed the study. Twenty-one participants (62%) used and returned the symptom diary. For 30 participants (88%), the app was installed correctly and twenty-five (83%) participants consistently used the app. We observed small, non-significant effects for taking adherence (*r* = 0.158) and timing adherence (*r* = 0.147), and small-medium (*r* = 0.294) for dosing adherence.

**Conclusion:**

This study demonstrated feasibility and fidelity of a smartphone-based therapy support in the community pharmacy setting. Trends suggest a potential improvement in adherence rates through smartphone reminders and text messages. These findings highlight the need for a fully powered trial.

## Highlights

• Community pharmacies can deliver high-quality app-based adherence support.
• The majority of patients demonstrated consistent engagement with the app.
• Apps show potential to support antibiotic therapies; larger studies are necessary.
• Targeted recruitment strategies are crucial for future adherence intervention studies.

## 1 Background

Antibiotics are among the most crucial medicines of our modern age.^1^ Between 2016 and 2018, the overall consumption of antibiotics worldwide ranged from 4.4 to 64.4 Defined Daily Doses (DDD) per 1000 inhabitants per day.^2^ In Switzerland, there are currently 309 different antibiotic products on the market.^3^ With 26% of all antibiotic prescriptions, amoxicillin/clavulanic acid was the most consumed antibiotic in Switzerland in the outpatient setting in 2022.^4^

One major challenge in antibiotic therapy is medication non-adherence, leading to subinhibitory antibiotic concentrations and consequently increasing the risk of resistance development.^5^ Growing antimicrobial resistances (AMR) remain a key problem of the antibiotic era. A systematic analysis from 2019 estimated 4.95 million deaths associated with AMR, of which 1.27 million were directly attributable to AMR.^5^ Besides mortality, AMR also increases the risk of recurring infections and prolonged hospitalization.^6^ In 2003, the World Health Organization (WHO) reported poor adherence as one major reason for drug resistance in the treatment of tuberculosis.^7^ Globally, adherence to antibiotic therapies is low, with an average of 62.2%.^8^ Prior research identified lower adherence as an independent risk factor for poor clinical outcome^9^ that can reduce treatment effectiveness and increase treatment failure.^10^, ^11^ For short-term antibiotic therapies, a non-adherence rate of 40% was observed in adult patients.^12^ Given that exact dosing and timing are crucial for therapy success,^13^ these findings highlight the potential impact of non-adherence on treatment outcomes.

In times of digitalization and mobile health, smartphone applications (apps) represent ubiquitous tools providing new possibilities for simple interventions to address non-adherence.^14^, ^15^ Various mobile health applications are available on the market with diverse content, functions, and quality.^16^ Most of them are intended to sustainably improve self-management and have been reported as useful in managing chronic diseases that require long-term medication.^17^, ^18^, ^19^ In contrast, little is known about their usefulness and effectiveness in short-term therapies. In 2021, a study showed that an adherence app with a reminder function was well accepted by patients undergoing short-term antibiotic treatment.^20^ However, the intervention's effect was not evaluated. Since forgetfulness is a significant barrier to adherence,^9^, ^21^ reminding patients to take their medication is a continuously pursued method of mobile health apps. This reminder function is often integrated in form of a sound and/or a push-message at scheduled intake times.^16^ A 2016 meta-analysis of 16 studies on chronic diseases reported a 36% increase in adherence with the use of an app-based reminder.^22^ An influence on clinical outcomes could not be demonstrated so far.^23^ While the potential of app-based reminders is known and demonstrated in long-term therapies, their potential in supporting adherence to short-term therapies is largely unexplored.

An additional factor contributing to poor adherence is a lack of knowledge or misunderstandings about the disease and/or medication.^21^, ^24^ To bridge these knowledge gaps, educating patients by providing therapy-related information is a promising approach to improving adherence.^14^ Thus, smartphones can serve as a simple tool that can combine both approaches - reminders and educational messages - in a single device. Additionally, some apps offer exportable files including an adherence report, which healthcare professionals (HCPs) can use to counsel patients on their treatment progress and adherence behavior.^16^

In recent years, the focus in healthcare research has gradually shifted from evaluating interventions solely for their effectiveness to considering how they can be successfully implemented in real-world practice.^25^ Implementation outcomes provide crucial insights into whether and how a service can be integrated into routine care.^26^ Among these, feasibility and fidelity are particularly important, as they help determine if an intervention can actually be delivered as intended and adopted in practice. Assessing these outcomes early during real-world testing offers valuable guidance for refining interventions and supporting their broader implementation.

With this pilot study, we aimed to evaluate the service and trial feasibility of a smartphone-based adherence support delivered by community pharmacists and to explore initial effects.

## 2 Methods

### 2.1 Study design

We performed a monocentric, cluster-randomized, double-blind, two-arm pilot trial conducted in a primary care setting in Northwestern and Central Switzerland. We invited 62 community pharmacies to participate in the study by e-mail or phone using a purposive sample strategy. Participating community pharmacies recruited eligible patients and offered the service during their daily practice. We included patients meeting the following eligibility criteria: older than 18 years of age; were prescribed an oral treatment with amoxicillin/clavulanic acid for 3–14 days; suffered from symptoms that correspond to a bacterial infection; accepted to use the smartphone application TOM™ during the study period; were capable of using the TOM™ application; signed the informed consent form; understood and spoke (Swiss) German; accepted to use a medication intake reminder. We excluded patients who obtained any motivational or educational inputs from apps on their smartphone to enhance medication intake (e.g. gamification, reward system, etc.), who did not manage their medication themselves, or who already used a medication intake reminder.

**Fig. 1:**
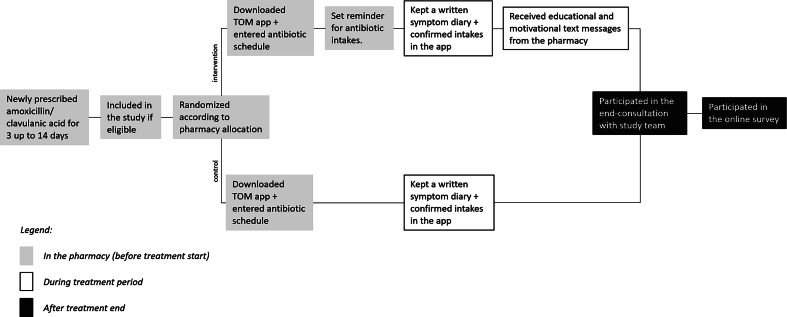
Overview of the study procedures.

Randomization took place at the community pharmacy level through block randomization with a block size of 4 assigning each community pharmacy to group 1 (intervention) or group 2 (usual care) and an allocation ratio of 1:1 (generated with www.randomizer.org). Allocation was performed by the study center before the initiation visit. Each community pharmacy recruited patients solely for their allocated group. Pharmacies and patients were not aware of the two-arm design of the study and were blinded to study group allocation. To measure adherence, all participants used a simplified version of the freely available medication management app TOM™ to confirm their medication intakes and kept a written symptom diary to assess intakes, symptoms or complaints, and their well-being once daily (see Fig. 1). The intervention group additionally received intake reminders and two text messages on the smartphone. Patients could choose their preferred intake reminder between the alarm or the calendar function of the smartphone or two freely available reminder apps. The first text message was educational, and the second was designed to motivate. Both messages were sent via e-mail or SMS by the pharmacist at pre-set time points during the therapy. The educational message was sent on the same day the antibiotic prescription was filled. The motivational message was sent halfway through the treatment period. The control group received no reminders and no text messages. One week after therapy ended, all participants obtained an end-consultation by phone with the study researcher (KM) to discuss their individual adherence data and symptom course. During the consultation, participants were asked about their current health status and any persisting symptoms. If adherence was suboptimal, participants were also asked about underlying reasons and potential difficulties with their medication use. Participants were then provided with guidance to optimize adherence, including information on the importance of regular intake and motivational advice. End-consultations were documented in written form using a predefined template. An online survey assessing satisfaction with the service using a 5-point Likert scale (1 = not satisfied to 5 = completely satisfied) was sent out after the consultation.

The initial target sample size was calculated based on adherence data from the literature.^22^ It was assumed that adherence in the control group would be 62%, and an app-based reminder could lead to a relative increase in adherence of 36%, resulting in an expected adherence rate of 84% in the intervention group. To detect this difference with 80% power and a 5% alpha risk, a total of 116 participants (58 per group) was required, accounting for 10% dropouts. However, due to recruitment challenges and a study duration preset at 12 months, we terminated the study before reaching the target sample size.

### 2.2 Data collection methods

TOM Medications™ is a freely available medication management app designed to support patients with their medication intake. For study purposes, a simplified, password-protected partner program was created within the app, which study participants could access once it was set up by the pharmacist. In the partner program, the medication and treatment regimen were entered together with the pharmacist. The program did not send intake reminders. The patient confirmed each planned intake manually. The TOM Medications™ app was selected because it could be customized in collaboration with the developers and configured as a simplified tool solely for medication intake tracking, without additional adherence-promoting features such as reminders, rewards, or motivational content.

The symptom diary assessed medication intakes (checkboxes for morning, midday, and evening), symptoms or complaints (free-text field), and overall well-being (5-point smiley scale; see **supplementary material Fig. S1**). The patient completed the diary once daily in the evening.

### 2.3 Evaluation of feasibility and fidelity

We evaluated the feasibility and fidelity of the service informed by recent guidelines.^27^ Therefore, we assessed recruitment rate of the study (defined as number of individuals who participated in the study compared to number of invited eligible patients), completion rate (defined as number of participants who completed the study compared to number of included participants), as well as accuracy and completeness of all data sources (case report forms, app data, data from diary). Reliability was assessed by calculating agreement between the diary and the app for each patient (defined as the proportion of measured intakes on which both methods agree, divided by the total number of measured intakes). Data from all participants who provided both measurement values were included. Additionally, we analyzed all performed end-consultations regarding statements toward usefulness of the service, perceived burden, or inconveniences and assessed the support needed with the delivery of the service (defined as number of contacts via the study hotline, phone or e-mail). We evaluated acceptability of the service via a 5-point rating scale for satisfaction in the online survey.

To measure fidelity (defined as the degree to which those responsible for delivering an intervention actually adhered to the intervention as outlined by its designers^28^, ^29^), we used observational measures. Therefore, we assessed whether the app was installed correctly, whether intake schedules were programmed as prescribed, and whether the intervention group received the scheduled text messages as intended. In addition, we assessed the extent to which the activities listed in the pharmacists' checklists within the case report form (CRF) were marked as completed.

### 2.4 Outcomes

As primary outcomes we defined the feasibility and fidelity indicators. Secondary outcomes were:

a) patients' satisfaction with the service (mean score);
b) proportion of days with well-being (percentage of days with green smiley rating compared to prescribed treatment days);
c) taking adherence (percentage of doses taken compared to prescribed doses);
d) timing adherence (percentage of doses taken within ±25% of the dosing time schedule compared to prescribed doses);
e) dosing adherence (percentage of days with correct number of doses taken compared to prescribed treatment days);
f) persistence (percentage of days with medication intake compared to prescribed treatment days);
g) standard deviation of dose-to-dose intervals (mean standard deviation of prescribed intervals);
h) proportion of days with symptoms (percentage of days with symptoms compared to prescribed treatment days);
i) type of used reminder systems (percentage of used reminder system types).

Adherence was calculated based on the time stamps of confirmed intakes in the TOM™ app and/or the diary. Analyses were performed based on app data. Missing data in one measurement method (app or diary) were imputed using the available value from the other method for further analysis. For the sensitivity analysis, we calculated the median of the highest and lowest values for each adherence outcome based on both measurement tools.

### 2.5 Statistical analysis

We calculated descriptive statistics for each group, by mean and standard deviation or median and interquartile range. Adherence was calculated at the individual level and expressed as a continuous percentage variable. As our data were not normally distributed, a non-parametric approach was applied. For exploratory analyses, we used the Mann-Whitney *U* test to compare both groups across all primary and secondary outcomes.

To estimate the magnitude of differences in adherence outcomes and revise power calculations for a fully powered study, we calculated the effect sizes r of the Mann-Whitney U test, which represents the standardized effect size for rank-based comparisons. Consistent with the non-parametric analytical strategy, the 95% confidence intervals were derived via bootstrapping with 1000 resamples.

Group comparisons were performed using R (version 4.4.2; 2024). A *p*-value of <0.05 was considered statistically significant.

## 3 Results

### 3.1 Participation

**Fig. 2:**
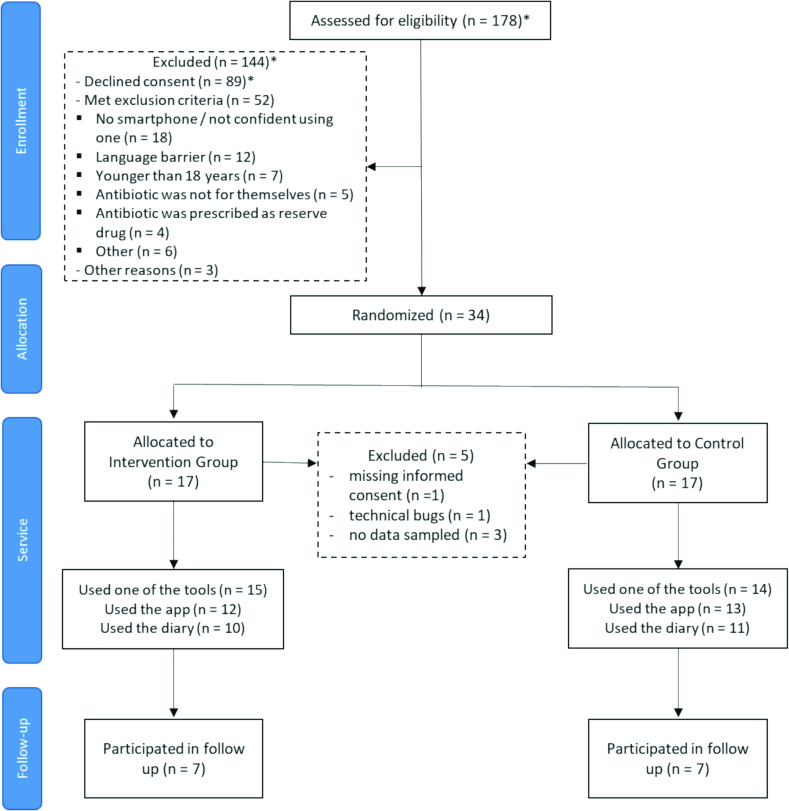
Participant flow chart illustrating the progression of participants throughout the study. Numbers marked with an asterisk represent estimates provided by recruiting pharmacists.

Between January 2024 and December 2024, a total of 57 community pharmacies participated in the study. A total of 18 community pharmacies recruited one or more patients; 39 community pharmacies could not recruit any patient. A total of 34 patients agreed to participate in the study (see Fig. 2). Participants were equally distributed among both groups with 17 participants each. There were no significant differences in demographics and clinical characteristics between participants in the intervention and control group (see Table 1). A total of 29 participants (85%; 15 participants in the intervention group, 14 participants in the control group) used at least one measurement tool that enabled us to calculate the primary outcome. A total of 21 participants (62%; 10 participants in the intervention group, 11 participants in the control group) kept a symptom diary, enabling us to calculate clinical outcomes (days with symptoms and days with well-being). Target sample size (*n* = 116) was not reached due to insufficient recruitment after the preset study duration of 12 months.

**Table 1:** Baseline demographics and clinical characteristics from the 34 recruited patients, presented per group. SD = standard deviation.

	Intervention group (*N* = 17)	Control group (*N* = 17)	p-value
Age [SD]	42.2 years [16.7]	37.1 years [12.7]	0.327
Sex	55.6% female 44.4% male	35.7% female 64.3% male	0.448
Treatment days [SD]	6.1 days [1.75]	6.9 days [1.56]	0.190
Dosing	38.9% 1000 mg 61.1% 625 mg	71.4% 1000 mg 28.6% 625 mg	0.140
Dosing regimen	33.3% 2× daily 66.7% 3× daily	57.1% 2× daily 42.9% 3× daily	0.323

### 3.2 Feasibility and fidelity indicators

A total of 123 eligible patients were invited to the study and 89 patients (72%) declined participation, indicating a recruitment rate of 28%. Twenty-nine out of 34 participating patients (85%) fully completed the study. A total of 21 out of the 34 participants (62%) used and returned the symptom diary. For 30 participants (88%), the app was correctly installed by the pharmacist. Three pharmacists incorrectly installed the full version of the app instead of the app version designed for the study. Due to technical bugs, the app could not be set up for one participant. Among the 30 participants with a correctly installed app, 25 (83%) consistently used the app to record their medication intakes over the treatment duration, indicating high adherence to the device. In the correctly installed apps, 97% of the intake schedules were programmed as prescribed. Overall, adherence rates calculated from the app data were lower than those calculated from symptom diaries (see **supplementary materials Table S1**). Mean agreement between app and diary was 87%, indicating a high level of concordance. All requested demographic data and clinical variables were fully documented in the CRF and all listed activities were checked by pharmacists in the CRF checklists. A total of 14 out of the 29 (48%) participants who completed the study participated in the end-consultation. During consultation, two participants in the intervention group reported that they did not receive any text messages.

The study team was not contacted by patients or pharmacists regarding problems with the delivery of the service. Contacts from pharmacists were limited to questions concerning study documentation. In the online survey, 9 out of the 13 participants (69%) indicated their satisfaction with the app and the service. None of the participants expressed dissatisfaction.

### 3.3 Secondary outcomes

Median taking adherence was 93% in the intervention group and 88% in the control group (see Table 2). Further median adherence rates ranged between 90% and 100% in the intervention group and 83% and 100% the control group. Median proportion of days with symptoms was 70% in the intervention group and 100% in the control group.

**Table 2:** Secondary outcomes given as median and interquartile range, if not mentioned otherwise; SD = standard deviation.

Outcome	Intervention group	Control group	p-value
Satisfaction [mean (SD)]	3.66 (0.52)	3.88 (0.75)	0.784
Proportion of days with well-being [%]	60 (40–100)	60 (50–92)	0.946
Taking adherence [%]	93.3 (90–100)	88.4 (81–95)	0.407
Dosing adherence [%]	100 (88–100)	85.7 (74–100)	0.118
Timing adherence [%]	90.0 (73–94)	83.3 (67–85)	0.479
Persistence [%]	100 (95–108)	100 (88–100)	0.587
SD dose-to-dose-intervals [h]	0.91 (0.2–2.4)	1.73 (0.9–2.4)	0.605
Proportion of days with symptoms [%]	70.0 (47–100)	100.0 (73–100)	0.254

There were no statistically significant differences in the outcomes between both groups. The effect sizes for taking and timing adherence were small with *r* = 0.158 (95% CI: 0.0048 to 0.49) and *r* = 0.147 (95% CI: 0.0088 to 0.52), respectively. The largest difference was observed in dosing adherence, with a 15% increase in the intervention group and a small-medium effect size of *r* = 0.294 (95% CI: 0.018 to 0.60). Patients in the intervention group primarily chose their smartphone's alarm clock as intake reminder (52.9%), followed by calendar function (29.4%) and one of the free apps (17.6%).

The sensitivity analysis revealed no deviation from the primary analysis. All results remained non-significant regardless of whether lowest or highest values were used (see **supplementary materials Table S2**).

## 4 Discussion

### 4.1 Principal results

This study aimed to investigate the feasibility and initial effects of a smartphone-based adherence support in short-term antibiotic therapy. Since over 80% of the participants completed the study and used the app to record their intakes, and pharmacy teams adhered to all planned procedures and delivered the intervention as intended, the overall feasibility and fidelity of the adherence service could be demonstrated. One issue encountered was that text messages were not sent in two cases. For similar future services, an automated sending system (e.g., directly integrated in the app) could be beneficial. Additionally, the low participation rate of 48% in the end-consultation suggests that this component of the adherence service may have been perceived as superfluous or uncomfortable by participants. Our feasibility findings are consistent with previous literature showing that mobile health interventions delivered by community pharmacies to support medication adherence demonstrate high feasibility and acceptability.^30^, ^31^, ^32^ Therefore, our results further support the growing body of evidence that both patients and pharmacists value the integration of digital tools into primary care to improve modern healthcare.

Overall, we observed high adherence rates in both study groups (intervention and control). With 88% taking adherence, the control group showed higher baseline adherence compared to the literature that informed our sample size calculation.^22^ Nevertheless, the results on the effect of the intervention indicate a tendency for increased adherence rates in the intervention group, although these differences were not statistically significant. Additionally, participants in the intervention group experienced fewer days with symptoms. Our findings align with previous studies that demonstrated a positive effect of smartphone reminders on medication adherence.^22^, ^33^, ^34^ However, the observed effect sizes in our pilot study were small whereas existing literature reported medium effect sizes for smartphone reminders.^33^ A possible explanation are the high adherence rates we observed in both groups, limiting the potential for improvement through the intervention. This high baseline adherence may be caused by the Hawthorne effect, meaning that simply using the app as a measurement tool and the feeling of being monitored had a positive influence on treatment adherence.

The clinical relevance of the observed adherence improvement in our study cannot be conclusively evaluated. Existing literature reports better clinical outcomes in antibiotic therapy when adherence is high and blood levels are optimal (e.g., through therapeutic drug monitoring).^9^, ^10^, ^35^ However, studies comparing clinical outcomes between patients with higher versus lower adherence reported lower baseline adherence levels and greater improvements in adherence than observed in our study. Therefore, the clinical impact of the smaller adherence improvement remains uncertain and could be further assessed in a larger, fully powered trial in the future.

Although our trial demonstrated general feasibility and fidelity, careful consideration of several key factors will be essential in similar future studies, especially when aiming for larger sample sizes. Overall, recruitment success in our study was limited and the target sample size could not be reached within the 12-month recruitment period. These recruitment difficulties might also be linked to the type of medication investigated. As most eligible patients were acutely ill due to their infection, the timing of the study inclusion process was suboptimal. The influence of health status on patients' willingness to participate in studies has already been described in the literature. In 2020, Gayet-Ageron et al. reported that patients who perceived themselves to be in good or excellent health were more willing to participate in clinical research than those declaring poor health.^36^ Additionally, the complexity of the intervention may have further contributed to low participation rates. In this study, we targeted all patients with antibiotic prescriptions, regardless of whether they experienced adherence difficulties, and offered a complex intervention requiring digital affinity and a significant time investment from both community pharmacy staff and patients. However, prior research suggests that the more complex an intervention, the more it targets individual patients who may have an explicit need for the intervention.^37^, ^38^ As such, we may have overestimated the recruitment potential as smartphone-based adherence services may be more appropriate for tech-savvy patients with suspected adherence issues rather than all patients with antibiotic treatment.^37^ Advertising such studies via online health platforms or apps from digital health insurances, with links to participating pharmacies, could be a promising approach to reach this target population.

As a factor facilitating recruitment of pharmacies, we observed that pharmacies with personal connections to the research team showed higher recruitment success. Highest recruitment success was achieved by the pharmacist who was involved in the study planning process, suggesting that engaging stakeholders in study design may be a key strategy to enhance commitment throughout the study. This aligns with current implementation science literature, which emphasizes the importance of stakeholder involvement at all stages of clinical trial design.^39^

### 4.2 Limitations

This study has several limitations. First, due to the small sample size, the results are of a descriptive nature and can only reveal trends and tendencies. Therefore, we could not draw final conclusions regarding an effect of a smartphone-based adherence service. To demonstrate a statistically significant improvement in adherence through an app-based service and assuming effect sizes of *r* = 0.158 for taking adherence and *r* = 0.294 for dosing adherence as observed in our pilot study, a fully powered trial (α = 0.05, power = 80%) would require participation of 496 patients per group with taking adherence or 143 per group with dosing adherence as the primary outcome.

Second, the comparison between adherence rates calculated from symptom diaries and those derived from app-based data revealed a tendency toward lower adherence reported via the app. During phone counseling, participants frequently reported that remembering to confirm medication intake in the app was more challenging than remembering the intake itself. Therefore, the app data may underestimate actual adherence to the antibiotic as the users' adherence to the device itself may have been low. This limitation might be mitigated by the fact that the app had the possibility to confirm intakes from the past. Accordingly, adherence measured by both tools was self-reported and may overestimate actual adherence due to social desirability and the Hawthorne effect.^40^ The sensitivity analysis further indicated that the calculated adherence rates varied considerably depending on the measurement method, suggesting limited robustness of the overall results and raising the question of which measure more accurately reflects true adherence. To minimize this risk of bias, future studies could rely on more objective adherence measurement tools instead of the diary as reference measure, such as medication event monitoring systems, where data cannot be self-modified by patients. However, the use of such systems would require significantly more resources, particularly in the context of a larger trial, as well as the system itself constitutes an additional unfamiliar tool for patients, which may reduce their willingness to participate in the study.

Third, we cannot exclude that participants in the intervention group silenced the reminder or that participants in the control group set their own reminder during the study period. By doing this, participants might have influenced the very essence of the intervention and thus, compromised the final results. However, we claim that participants would have mentioned such deep changes during the end-consultation.

Fourth, we cannot rule out the possibility of selection bias. Patients who are willing to participate in the studies may be more likely to adhere to their intake schedules, leading to overestimated adherence rates in comparison to the general population.^40^, ^41^ Although not statistically significant, our data suggest demographic differences between the two groups, particularly in sex distribution and dosing frequency. This situation may further limit the generalizability of our findings. For instance, previous studies suggest that women tend to have lower adherence rates than men.^42^, ^43^ Additionally, missing a single dose in a three-times-daily regimen has smaller impact on calculated adherence than missing a dose in a twice-daily regimen, especially over a short treatment period. These differences may therefore have influenced the observed adherence rates in both groups.

Fifth, a considerable proportion of the participants did not provide complete datasets. The missing data were likely not completely at random. It is plausible that participants with lower adherence to the intervention were also less adherent to device use or diary completion. Furthermore, a substantial proportion of responses was missing in the assessment of participant satisfaction. This may have introduced attrition bias and could have resulted in an overestimation of feasibility, participant satisfaction, and adherence-related outcomes.

Sixth, feasibility and fidelity were mainly assessed through observational measures of the trial process and service delivery, as well as patients' satisfaction with the service. However, the perspective of the participating pharmacists as service providers and trial deliverers is missing in this project for a more comprehensive evaluation. Gathering their perspective, for example through interviews, could have offered deeper insights into factors that influenced service and trial feasibility and fidelity. Moreover, several aspects related to patient engagement and usability were not explored in this study. For example, participants' education levels and digital skills were not assessed, which may have influenced their interaction with the app and adherence to the intervention. Additionally, satisfaction with the app interface itself was not evaluated, limiting insights into usability and user experience. Addressing these aspects in future studies could provide a more comprehensive understanding of factors affecting feasibility and fidelity of an app-based adherence support.

### 4.3 Strengths

One of the key strengths of this study is its double-blind, cluster-randomized controlled trial design. The randomization helped distribute known and unknown participant- and site-level factors across both groups, reducing the risk that outcome differences were due to baseline imbalances rather than the intervention itself. However, given the small sample size, this advantage should be interpreted cautiously, as chance imbalances between groups remain possible as observed for sex distribution and dosing. Double-blinding of pharmacists and patients minimized potential performance bias, such as differential motivation or behavior in the intervention versus control group, thereby supporting the internal validity of our study findings. A second key strength is the real-world setting in which the study was conducted. By recruiting participants directly in the community pharmacy, we were able to assess not only clinical effects but also the potential for the implementation of such an adherence service in practice as recommended by implementation sciences.^25^ Our results may inform the design of a future fully powered effectiveness study, which can draw on the estimated effect sizes for power calculation and consider our observations related to the service and trial implementation.

## 5 Conclusion

The smartphone-based adherence support indicated small effects on adherence improvement in short-term antibiotic therapies. However, these effects were not statistically significant due to the limited sample size. The pharmacists' ability to recruit patients during their daily practice, along with the high quality of the sampled data and study materials, demonstrated the feasibility of conducting such a study in a community pharmacy setting. Nevertheless, a larger-scale study with a higher sample size is needed to confirm the observed effects. Effective strategies for recruitment will be essential. Involving pharmacies and patients in the study design could be one such strategy to enhance recruitment success.

## CRediT authorship contribution statement

**Kirstin Messner:** Writing – original draft, Project administration, Conceptualization. **Vanessa Sutter:** Writing – review & editing, Project administration, Conceptualization. **Samuel Allemann:** Writing – review & editing, Supervision, Conceptualization. **Isabelle Arnet:** Writing – review & editing, Supervision, Project administration, Conceptualization.

## Ethical considerations

This study was approved by the Ethics Committee Nordwest- und Zentralschweiz (No. 2023–01753) on November 21, 2023. All participants provided written informed consent prior to enrolment in the study. This research was conducted ethically in accordance with the World Medical Association Declaration of Helsinki.

## Funding

This research did not receive any specific grant from funding agencies in the public, commercial, or not-for-profit sectors.

## Declaration of competing interest

The authors declare that they have no known competing financial interests or personal relationships that could have appeared to influence the work reported in this paper.

## Data Availability

The datasets generated and analyzed in this study are not publicly available but are available from the corresponding author on reasonable request.
