# Supplementary material for: Feasibility, fidelity and initial effects of an app-based service for short-term antibiotic therapy: A pilot study in a primary care setting
Source: Explor Res Clin Soc Pharm. 2026 Apr 21;23:100790. doi: 10.1016/j.rcsop.2026.100790 (PMC13141640; doi:10.1016/j.rcsop.2026.100790)

**Figure S1.** Symptom diary used by participants to track medication adherence, symptoms/complaints, and well-being.


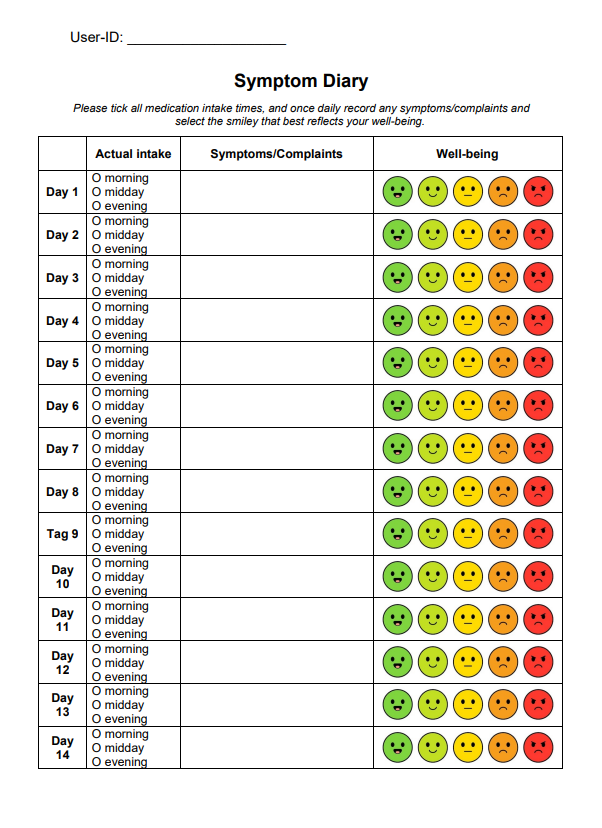

Supplement: Supplementary file 1 — Supplementary material 1 [file mmc1.docx]
