# Supplementary material for: Feasibility, fidelity and initial effects of an app-based service for short-term antibiotic therapy: A pilot study in a primary care setting
Source: Explor Res Clin Soc Pharm. 2026 Apr 21;23:100790. doi: 10.1016/j.rcsop.2026.100790 (PMC13141640; doi:10.1016/j.rcsop.2026.100790)
Supplement: Supplementary file 2 — Supplementary material 2 [file mmc2.docx]

**Table S1:** Influence of the chosen adherence measurement tool on adherence and persistence rates, given as median and IQR.

| **Parameters** | **Taking adherence [%]** | **Dosing adherence [%]** | **Persistence [%]** |
| --- | --- | --- | --- |
| Intervention group | | | |
| Combined values | 93.3 (90-100) | 100 (88-100) | 100 (95-108) |
| App values | 91.7 (88-94) | 100 (85-100) | 100 (88-104) |
| Diary values | 96.7 (83-100) | 100 (90-100) | 100 (90-100) |
| Control group | | | |
| Combined values | 88.4 (81-95) | 84.5 (74-100) | 100 (88-100) |
| App values | 85.0 (80-94) | 83.3 (71-100) | 100 (83-100) |
| Diary values | 100 (98-107) | 100 (95-100) | 100 (100-116) |
