# Supplementary material for: Feasibility, fidelity and initial effects of an app-based service for short-term antibiotic therapy: A pilot study in a primary care setting
Source: Explor Res Clin Soc Pharm. 2026 Apr 21;23:100790. doi: 10.1016/j.rcsop.2026.100790 (PMC13141640; doi:10.1016/j.rcsop.2026.100790)
Supplement: Supplementary file 3 — Supplementary material 3 [file mmc3.docx]

**Table S2:** Sensitivity analysis: Influence of the highest and lowest adherence measurement values on primary and secondary outcomes. Outcomes are given as median and IQR.

| **Outcome** | **Intervention** | **Control** | **p-value** |
| --- | --- | --- | --- |
| Highest values | | | |
| Taking adherence [%] | 93.3 (91-100) | 100 (92-108) | 0.402 |
| Dosing adherence [%] | 100 (100-100) | 100 (84-100) | 0.389 |
| Persistence [%] | 100 (100-108) | 100 (100-116) | 0.796 |
| Lowest values | | | |
| Taking adherence [%] | 91.7 (87-98) | 87.5 (81-94) | 0.370 |
| Dosing adherence [%] | 100 (85-100) | 84.5 (74-98) | 0.124 |
| Persistence [%] | 100 (87-100) | 100 (84-100) | 0.391 |
